# ^13^CO_2_ labelling: new uses for a well-established photosynthesis research tool

**DOI:** 10.1093/jxb/eraf370

**Published:** 2025-10-09

**Authors:** Vittoria Clapero

**Affiliations:** Max-Planck-Institute of Molecular Plant Physiology, Am Mühlenberg 1, D-14476 Golm, Germany

**Keywords:** CO_2_ labelling, drought stress, leaf heterogeneity

## Abstract

This article comments on:

**Liu J, Zhao J, Ling X, Xiong D**. 2025. ^13^C labeling to determine intra-leaf photosynthetic heterogeneity dynamics during drought and rewatering. Journal of Experimental Botany **76**, 4640–4653. https://doi.org/10.1093/jxb/eraf215

This article comments on:

**Liu J, Zhao J, Ling X, Xiong D**. 2025. ^13^C labeling to determine intra-leaf photosynthetic heterogeneity dynamics during drought and rewatering. Journal of Experimental Botany **76**, 4640–4653. https://doi.org/10.1093/jxb/eraf215

**The study of carbon incorporation in plants has been the cornerstone of photosynthesis research since its beginning, with the supply of isotopically labelled CO_2_ being one of the fundamental methods employed. The discovery of the photosynthetic pathway itself was supported by labelling in the early stages (Hatch and Slack, 1966). Back then, however, labelling was carried out using unstable radioactive ^14^C which presents several drawbacks: it is a hazardous substance, difficult to dispose of, and requires dedicated laboratories and equipment for handling (Baccolini and Arrivault, 2024). A different approach to carbon labelling has been established that deals with these drawbacks: labelling CO_2_ with the heavier stable and non-radioactive ^13^C isotope (Box 1). The quantification of incorporated ^13^C via MS has allowed us to study the flux of carbon into a large number of metabolites and has been used to gain useful knowledge about differences between C_3_ and C_4_ photosynthesis (Treves *et al*., 2022; Clapero *et al*., 2024), as well supporting the genetic engineering efforts aimed at improving photosynthetic efficiency (Ermakova *et al*., 2021).**

Liu *et al*. (2025) describe a new application for ^13^CO_2_ labelling by studying heterogeneity of photosynthetic recovery following drought along a maize leaf blade. To track heterogeneity using their newly developed method, Liu and colleagues switch briefly (∼1.5 min) to the heavier isotope ^13^CO_2_ as the sole source of CO_2_ across a fully expanded assimilating maize leaf blade. The resulting ^13^C incorporation enabled the authors to precisely estimate photosynthetic rates simultaneously at several points along the leaf blade, improving what was previously feasible to achieve in studies of leaf heterogeneity with gas exchange, fluorescence, and thermal imaging. The results from their new system demonstrate the sensitivity of leaf tips to drought and challenge the convention of using spot measurements at the middle of the leaf as representative of whole-leaf responses.

Box 1. **Carbon isotope discrimination and photosynthesis**

Isotope labelling happens naturally during photosynthesis. Carbon occurs in nature as two stable isotopes, the lighter ^12^C and the heavier ^13^C. However, ^13^C is quite rare, and in the atmosphere ^13^CO_2_ represents only 1.1% of all CO_2_ (Farquhar *et al*., 1989). The relative abundance of ^13^C to ^12^C in plants is even lower than in the air, indicating that plants preferentially fix ^12^CO_2_. The stable carbon isotope ratio (indicated with δ^13^C) of a sample is expressed as the ^13^C/^12^C ratio of said sample (R_S_) in reference to the ^13^C/^12^C of a standard (R_PDB_), which conventionally has been the Pee Dee Belemnite Standard, a fossil with an exceptionally high amount of the ^13^C isotope (Farquhar *et al*., 1982).

$$\delta^{13}C=\;\frac{R_{S}-R_{\mathrm{PDB}}}{R_{\mathrm{PDB}}}(\text{‰})$$

The difference between the δ^13^C of a plant sample and the surrounding air is termed carbon isotope discrimination (Δ^13^C, Farquhar *et al*., 1982):

$$\Delta^{13}C=\;\frac{\delta^{13}C_{\mathrm{air}\;}-\delta^{13}C_{\mathrm{plant}}}{1+\;\delta^{13}C_{\mathrm{air}}}$$

Plants discriminate against ^13^C during carbon assimilation and therefore have more negative values than the air. It was theorized (Farquhar and Richards, 1984) and later extensively proven in a variety of species that a lower discrimination against ^13^C is directly connected to a higher water use efficiency (WUE; biomass produced per unit of water transpired), as they both depend on stomatal conductance and CO_2_ assimilation rates (see review by Eggels *et al*., 2021). While in C_3_ plants the isotopic discrimination is mostly driven by the preference of Rubisco for ^12^C, in C_4_ plants this relationship is complicated by the various steps of the carbon concentration mechanism involving mesophyll and bundle sheath cells (von Caemmerer *et al*., 2014). Therefore, the correlation between Δ^13^C and WUE is more direct in C_3_ plants, even though correlations can also be drawn in C_4_ species (Ellsworth and Cousin, 2016; Eggels *et al*., 2021). The variation of Δ^13^C along the leaf length could therefore also provide information about WUE heterogeneity, but lacks the temporal resolution (Ellsworth and Cousin, 2016) that could be achieved with labelling in the study of Liu *et al*. (2025) (i.e. before and after drought). Nevertheless, using Δ^13^C as a proxy for wide-range screening of drought-resistant accessions remains a viable strategy, especially considering the costs of pure ^13^C labelling, which can be prohibitively expensive.

## Leaf heterogeneity of monocots

Leaves are complex organs, and several studies have pointed out how different areas along the blade of narrow-leafed plants such as monocots (e.g. maize and rice) can display radically different characteristics. Leaf development and plant architecture of monocot grasses strongly impact base-to-tip differences, as the leaf cells develop in linear ranks from tip to base (Conklin *et al*., 2019; Strable and Nellissen, 2021), and the basal portion of the leaves is usually more shaded than the central and terminal parts (Wang *et al*, 2014; Xiong *et al*., 2015). This results in multiple and intertwined differences, for example gradients of stomatal density, nitrogen, and Rubisco content, that ultimately affect the variation in photosynthetic efficiency and source–sink relationships along the leaf blade (Wang *et al*., 2014; Xiong *et al*., 2015). In addition to the biochemical level, clear gradients can also be observed by studying the abundance of transcripts along the leaf blades of rice and maize (Pick *et al*., 2011; Wang *et al*., 2014). Indeed, gene regulation may also change along the leaf blade, as was recently shown in rice (Robertson and Wilkins, 2023). The finding by Liu *et al*. (2025) that photosynthetic capacity increases from leaf base to tip in maize is therefore not surprising. However, their work advances on this observation by realizing that narrow leaves are also heterogeneously affected by stresses such as drought (being prone to tip desiccation), leading Liu *et al*. (2025) to hypothesize that this would result in post-desiccation photosynthetic recovery heterogeneity. Their demonstration of the base to tip gradient in drought recovery potential is highly novel and in accordance with previous scientific literature. Further understanding of the extent of intra-leaf trait variation is crucial for accurate interpretation of plant research outcomes that could be misjudged if leaf heterogeneity is not considered and the specific leaf portion measured is not taken into account. For example, measurements such as gas exchange are often performed on the middle part of the leaf, and, as the authors explain, this could lead to erroneous estimation of overall photosynthetic rates or drought sensitivity, ultimately impacting not only on the reproducibility of the results but also their overall correct interpretation. Not only the position along the leaf blade but also the leaf position on the plant should be carefully considered, as older leaves on the lower parts of the plant seemed to be more affected by drought in the study by Liu *et al*., reducing the overall photosynthetic recovery capacity. As the authors point out, such heterogeneity is often encountered in the experimental set-up but not reported. These findings are also relevant for simulation models that typically assume uniform leaf and plant characteristics and could therefore overestimate the photosynthetic recovery capacity at the canopy level, not accurately reflecting the complexity of leaf responses. Nevertheless, the real impact of leaf tip sensitivity to desiccation on whole-plant performance will ultimately depend on the leaf area lost and could be negligible considering the high leaf area index (canopy area to ground area ratio) of many grasses.

## Drought stress is particularly detrimental for photosynthesis

Water constitutes up to 90% of a plant leaf biomass (Erice *et al*., 2018), making drought stress a severe threat to plant productivity. The usual cause of drought stress is insufficient rainfall, although increased soil evapotranspiration due to high temperatures and salinity stress can also lead to reduced water availability for plants (Yin *et al*., 2018; Yang *et al*., 2019; Cohen *et al*., 2021). Drought stress is therefore a looming possibility for many crops given the reality of global warming and rainfall anomalies experienced around the world (Ray *et al*., 2019; Konapala *et al*., 2020).

All life stages of a plant can be affected, as drought not only negatively impacts seed germination and vigour (Hussain *et al*., 2018; Panda *et al*., 2021; Abreha *et al*., 2022), but also affects overall plant growth, leaf number, and area, among others (Farooq *et al*., 2009; Yang *et al*., 2021). The effect of drought on yield is markedly negative and, based on the timing, can affect different yield components throughout crop ontogeny, as it can shorten the grain-filling period in cereals, reduce the number of harvested reproductive organs, and also induce infertility in flowers or seed abortion (reviewed in Farooq *et al*., 2009; Dietz *et al*., 2021). The article by Liu *et al*. (2025) sheds further light on the detrimental impact that drought has on maize leaves, focusing on photosynthetic recovery. Photosynthesis is one of the main metabolic processes affected by drought, mostly because of restricted CO_2_ availability due to stomatal closure. Minimization of water loss to transpiration is mediated by the phytohormone abscisic acid (ABA) and represents an important trait for drought tolerance (Dietz *et al*., 2021), with swift stomatal movements being directly linked to water use efficiency (Lawson and Vialet-Chabrand, 2019). However, stomatal closure exposes the leaves to photodamage and reactive oxygen species (ROS) damage, in turn causing disruption in the thylakoid membrane (Laxa *et al*., 2019; Yang *et al*., 2021). There are also non-stomatal factors involved in the reduction of photosynthesis under drought, such as, for example, reduction of both Rubisco activity and its regeneration process, and diminished activity of other photosynthetic enzymes (reviewed in Farooq *et al*., 2009; Zargar *et al*., 2017). The leaf levels of photosynthetic and accessory pigments—chlorophylls and carotenoids—are also reduced under drought regimens (Yang *et al*., 2021). Stomatal limitation in particular has an impact on plant performance in a drought–rewatering cycle, being the key determinant of photosynthetic recovery capacity in maize leaves.

**Fig. 1.**
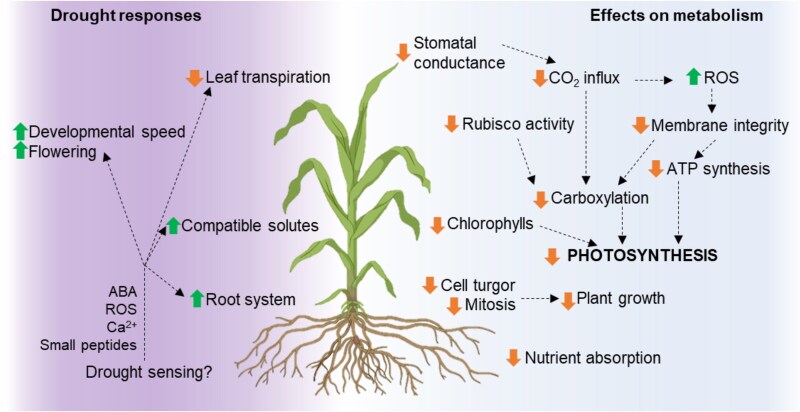
Effects of drought on plant metabolism and coping strategies. Drought is suggested to be first sensed in the roots and communicated to the rest of the plant via Ca^2+^, ROS, ABA, and small peptide signalling. Drought responses are manifold (left half); for example, plants can escape drought by increasing developmental speed in combination with early flowering, invest in their root system to maintain soil water uptake, synthesize compatible solutes to maintain the osmotic potential, and decrease their leaf transpiration by reducing stomatal conductance (reviewed in Farooq *et al*., 2009; Kuromori *et al*., 2022; Yang *et al*., 2021). Stomatal closure in turn starts a cascade of effects on metabolism (right half), causing reduced CO_2_ influx and ROS stress in the chloroplast, negatively affecting photosynthesis (see text). Processes stimulated by drought are indicated with upwards arrows, and processes negatively affected by drought are indicated by downwards arrows. Figure created in BioRender, Clapero, V. (2025) https://BioRender.com/0p3x574.

Considering the multifaceted effect of drought on photosynthesis (Fig. 1) and the imminent loss of crop yield to drought, the work by Liu *et al*. (2025) is important to uncover the heightened sensitivity to drought of the most photosynthetically efficient part of the leaf, which may feed into research and breeding efforts to stabilize crop yield under water-limited conditions. While the mechanism underlying the heightened sensitivity of the leaf tip is not fully elucidated, the authors hypothesize that reduced water storage capacity and/or high embolism vulnerability in the tip are most probably responsible for the observed phenotype, providing a clear starting point for further experimental validation. In addition, the simultaneous sampling procedure along the leaf blade for photosynthetic rate estimation would be perfectly suited to be combined with molecular techniques such as (single-cell) RNA-seq to take a look into the genetic basis of the observed phenotypic heterogeneity (Kakumanu *et al*., 2012; Xu *et al*., 2021).

## Data Availability

Data is publicly available.
